# Increased PTP1B expression and phosphatase activity in colorectal cancer results in a more invasive phenotype and worse patient outcome

**DOI:** 10.18632/oncotarget.7829

**Published:** 2016-03-01

**Authors:** Elmer Hoekstra, Asha M. Das, Marloes Swets, Wanlu Cao, C. Janneke van der Woude, Marco J. Bruno, Maikel P. Peppelenbosch, Peter J.K. Kuppen, Timo L.M. ten Hagen, Gwenny M. Fuhler

**Affiliations:** ^1^ Department of Gastroenterology and Hepatology, Erasmus MC, University Medical Center Rotterdam, Rotterdam, The Netherlands; ^2^ Department of Surgery, Section Surgical Oncology, Laboratory Experimental Surgical Oncology, Erasmus MC, University Medical Center Rotterdam, Rotterdam, The Netherlands; ^3^ Department of Surgery, Leiden University Medical Center, Leiden, The Netherlands

**Keywords:** colorectal cancer, kinases and phosphatases, biomarker, metastasis

## Abstract

Cell signaling is dependent on the balance between phosphorylation of proteins by kinases and dephosphorylation by phosphatases. This balance if often disrupted in colorectal cancer (CRC), leading to increased cell proliferation and invasion. For many years research has focused on the role of kinases as potential oncogenes in cancer, while phosphatases were commonly assumed to be tumor suppressive. However, this dogma is currently changing as phosphatases have also been shown to induce cancer growth. One of these phosphatases is protein tyrosine phosphatase 1B (PTP1B). Here we report that the expression of PTP1B is increased in colorectal cancer as compared to normal tissue, and that the intrinsic enzymatic activity of the protein is also enhanced. This suggests a role for PTP1B phosphatase activity in CRC formation and progression. Furthermore, we found that increased PTP1B expression is correlated to a worse patient survival and is an independent prognostic marker for overall survival and disease free survival. Knocking down PTP1B in CRC cell lines results in a less invasive phenotype with lower adhesion, migration and proliferation capabilities. Together, these results suggest that inhibition of PTP1B activity is a promising new target in the treatment of colorectal cancer and the prevention of metastasis.

## INTRODUCTION

Colorectal cancer (CRC) remains a global health burden, even though treatment strategies have improved significantly over the last decades. At the time of diagnosis, approximately 20% of patients already present with metastasis, dropping their 5 year-survival from 90% to a mere 12% [1]. Therefore adjuvant therapy is now focused on avoiding the transition into this metastatic state.

Tyrosine phosphorylation is a well-established post-translational mechanism by which normal cellular homeostasis is conserved. This process is orchestrated by two opposing enzymes; kinases which phosphorylate tyrosine residues on target proteins, and phosphatases which catalyze the hydrolysis of these phosphoester bonds. Unbalanced tyrosine phosphorylation, as observed in many cancers, results in dysregulation of pro-tumorigenic processes such as cell proliferation, adhesion, and migration [2]. Kinases are known to be activators of oncogenic signaling, and kinase inhibitors have been developed with promising clinical success [3–5]. The equally important phosphatases have so far not been acknowledged as potential targets for treatment. However, it is slowly becoming clear that in addition to counteracting kinase activity, phosphatases may also act as initiators of signaling themselves, making them an interesting new focus in cancer research [6]. One of these dual function enzymes is the protein tyrosine phosphatase 1B (PTP1B), encoded by the *PTPN1* gene; a ubiquitously expressed non-receptor protein tyrosine phosphatase located on the cytoplasmic face of the endoplasmic reticulum. It was the first phosphatase to be discovered in the 1980s, and is often referred to as the prototypical non-receptor PTP [7].

Although the role of PTP1B as negative regulator in diabetes signaling is well established [8], the role for this enzyme in cancer is controversial. It has been implicated in dephosphorylating various growth factor receptors, such as the epidermal growth factor receptor (EGFR) [9], platelet derived growth factor receptor (PDGFR) [10] and insulin receptor (IR) [11], as well as cytoplasmic kinases like Src [12], Bcr/Abl and JAK2 [13]. Many of these substrates possess oncogenic properties when activated through phosphorylation, which makes it reasonable to suggest that PTP1B acts as a tumor suppressor by dephosphorylation of these proteins. In line with this theory it has been shown that P53 and PTP1B double knockout mice display an increased number of B cell lymphomas and thereby decreased survival as compared to the P53-knockout mice alone [14].

In contrast, there is also literature showing that PTP1B might act as a tumor promoter. Wang and colleagues have shown both *in vivo* and *in vitro* that PTP1B is upregulated in gastric cancer and is associated with increased tumorigenicity and metastasis [15]. Similarly, Lessard et al. propose a tumor promoting role for this phosphatase in prostate cancer, with its expression correlating to that of the androgen receptor [16]. Most extensively studied however, is the role of PTP1B as tumor promoter in breast cancer, as a strong correlation is found between PTP1B and HER2 (ErBb2) overexpression [17]. Indeed, PTP1B deficiency in HER2-activated mouse models leads to a significant delay in tumor progression and prevents metastasis [18], whereas overexpression of PTP1B in mammary glands of mice induces tumorigenesis, suggesting that enhanced PTP1B in itself is enough to confer breast cancer potential [17]. Moreover, it was recently shown that the specific PTP1B inhibitor Trodusquemine (MSI-1436) inhibits tumor formation and completely abrogates metastasis in breast cancer mouse models [19]. A tumor promoting role for PTP1B in colorectal cancer (CRC) has also been proposed [20, 21], although its contribution to cellular cancer hallmarks and signaling remains unclear. This study aims to further elucidate the role of PTP1B phosphatase in CRC, focusing on its expression levels, and arguably of more importance, the intrinsic phosphatase activity of this enzyme.

## RESULTS

### *PTPN1* gene expression is increased in colorectal cancer

**Figure 1:**
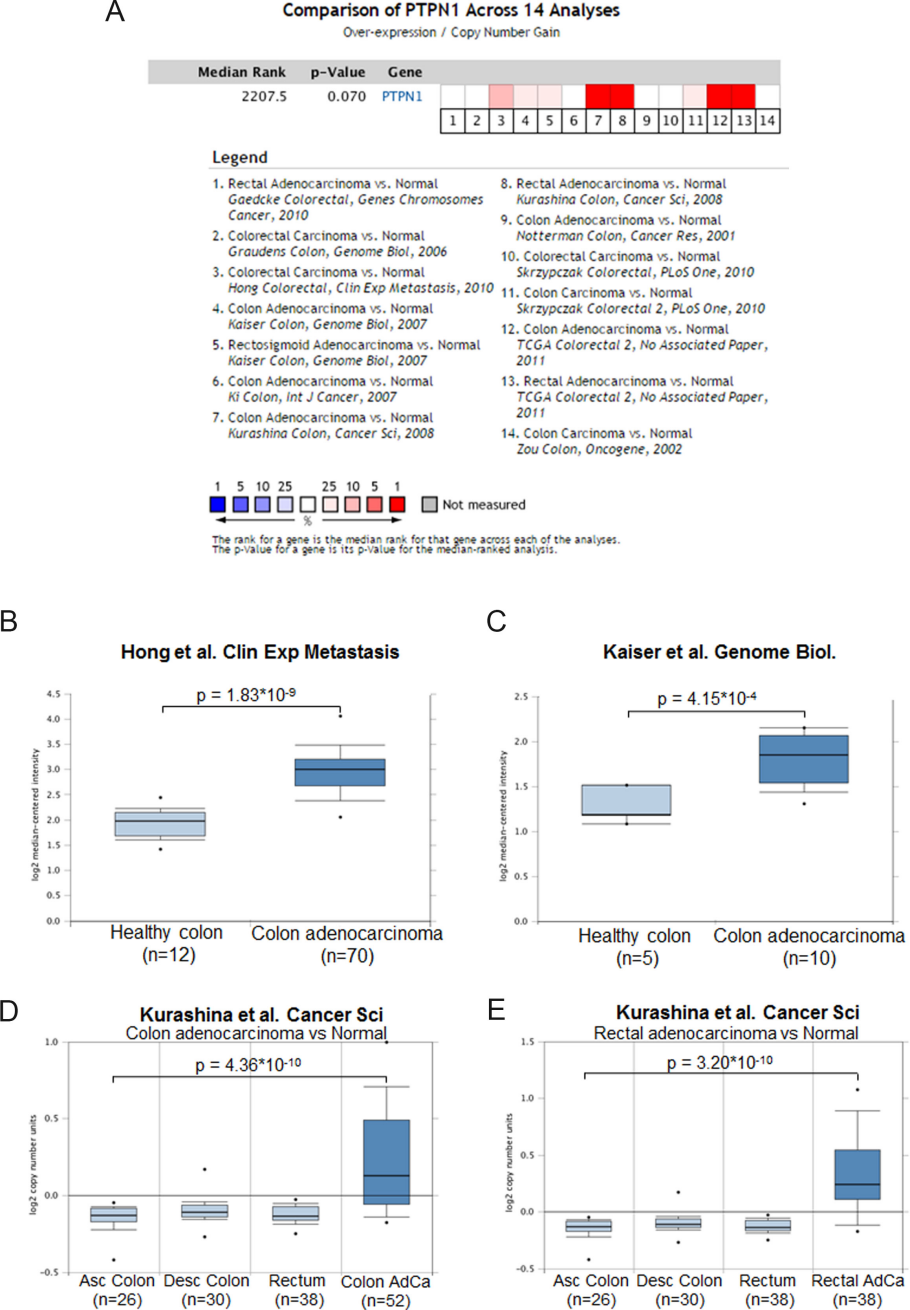
PTPN1 expression is increased in colorectal cancer **A.** Oncomine analysis of the colorectal cancer datasets reporting on *PTPN1* expression shows that in 8 out of 14 datasets report an overexpression of *PTPN1* in cancer as compared to normal colonic tissue. **B-E.** Representation of individual datasets reporting on *PTPN1* expression from oncomine website, analyzed using unpaired students' T-test.

To get a better understanding of the role of PTP1B in colorectal cancer, we first investigated expression levels of the gene encoding this phosphatase (*PTPN1*) using publicly available array data sources. The Oncomine Cancer Microarray database (http://www.oncomine.org/) contains 14 datasets comparing human colorectal cancer to their normal tissue counterparts. As shown in Figure 1A, 8 of these 14 datasets show a significant *PTPN1* upregulation in cancer as compared to normal tissue, with fold changes ranging from 1.27 [26] to 2.011 [27] (Figure 1B–1E). The TCGA Colorectal 2 dataset compares normal colon to 7 subtypes of colorectal cancer, and shows a *PTPN1* copynumber increase for 6 of these cancer types (Figure S1A, S1B). Using cbioportal, we further analyzed the TCGA colorectal cancer dataset [28], revealing an alteration in the *PTPN1* gene (e.g. amplification, mutation, upregulation) in 45.1% of cases (Figure S1C) and a trend towards worse survival for patients with alterations in the *PTPN1* gene (Figure S1D). No correlation was observed between *PTPN1* expression and DNA methylation, suggesting that the observed upregulation is not mediated by an aberrant methylation pattern. Network analysis of the *PTPN1* gene associations in colorectal cancer shows links with several oncogenes such as EGFR, STATs, and Src (Figure S1E).

### PTP1B protein is overexpressed in primary colorectal cancer samples

**Figure 2:**
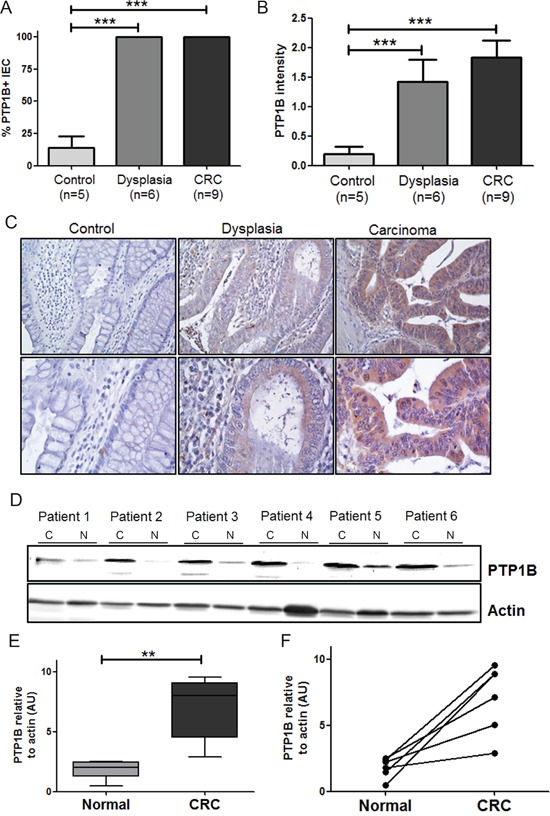
Expression levels of PTP1B are follow a stepwise increase from control, to dysplasia, to carcinoma Tissues of patients with inactive ulcerative colitis (Control, n=9), dysplasia (n=5), and colorectal cancer (CRC, n=7) were stained for PTP1B by immunohistochemistry. PTP1B staining was scored for percentage of positive intestinal epithelial cells **A.** as well as intensity of staining **B.** and statistical analysis was performed using Mann-Whitney t-test. (*** P >0.001). **C.** Representative examples (10x and 40x magnifications) of control, dysplasia, and CRC are shown. **D.** Western blot analysis of PTP1B expression in 6 paired freshly frozen colorectal cancer; C and normal adjacent tissues; N, with β-actin as loading control. **E.** Quantification of western blot represented as means or individual pairs (** P >0.01). **F.** Same data as in E, but individual patients shown. Bars connect CRC samples to their corresponding.

Next, we investigated whether the observed increase in *PTPN1* expression corresponds to increased PTP1B protein levels in colorectal dysplasia and carcinoma. Immunohistochemistry was performed on microsections of biopsies of low/high grade dysplastic polyps and adenomas (dysplasia; n=6), adenocarcinoma (n=9) and controls (n=5). PTP1B expression was apparent in the cytoplasm of intestinal epithelial cells (IEC), and the intensity of the staining followed a step-wise increase from untransformed tissue to dysplasia and carcinoma (Figure 2A–2C). Using a different technique, we confirmed PTP1B protein overexpression in 6 paired freshly frozen tumor and normal adjacent tissues by Western blotting, demonstrating a significant increase in the total levels of this phosphatase in the tumor tissue (Figure 2D–2F).

### Relation PTP1B with patient outcome

**Figure 3:**
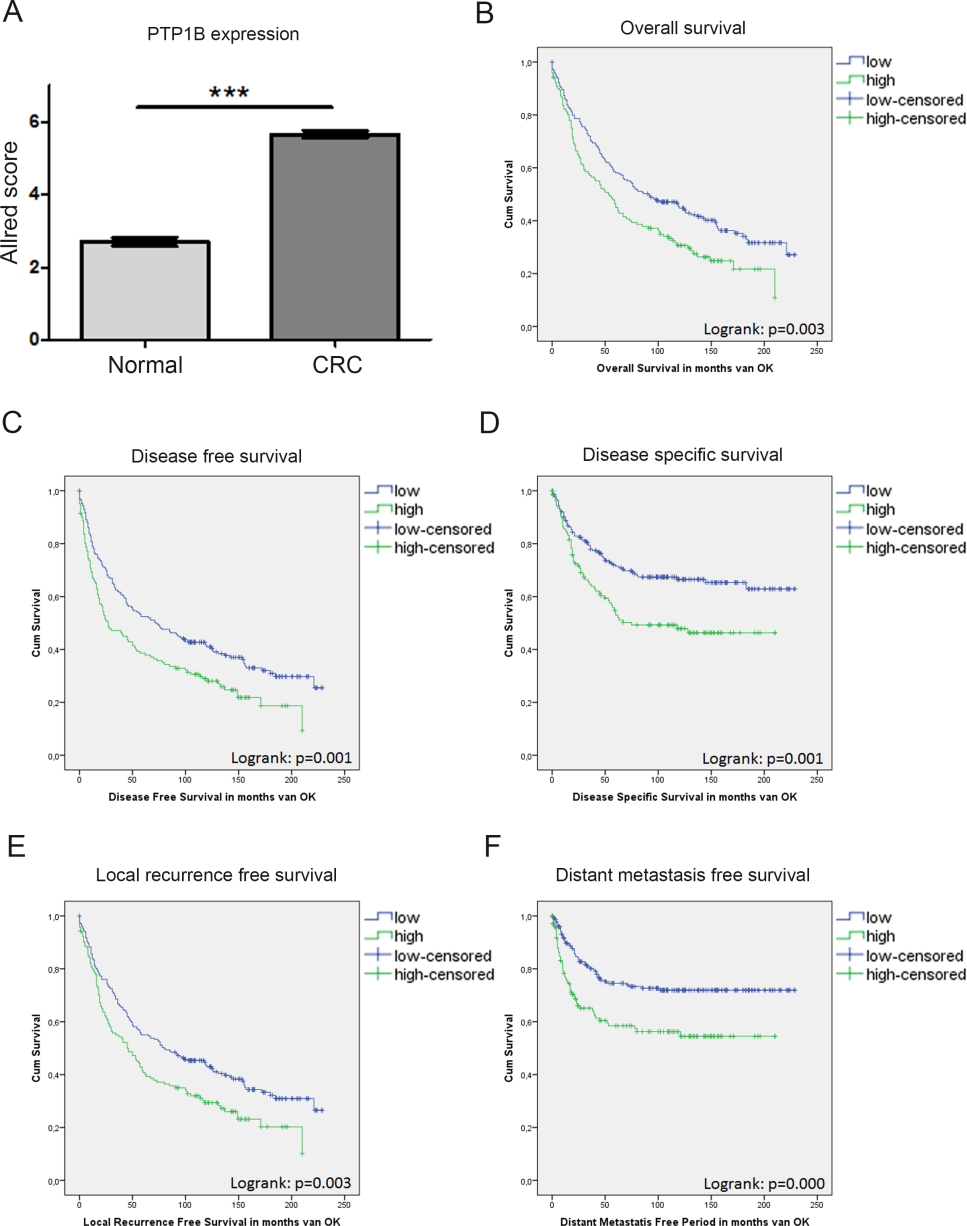
PTP1B expression in a large cohort of CRC patients is correlated to a worse patient survival IHC analysis of PTP1B on a tissue micro array (TMA) of colorectal cancer patients (n=371) and healthy adjacent tissue (n=251) using the Allred score. **A.** Allred score is significantly increased in CRC compared to control. Patients are divided in two groups based on the Allred score (Low < 6; High >6). **B.-F.** Kaplan meier curves for overall survival, disease free survival, disease specific survival, local recurrence free survival, and distant metastasis free survival reveal. High PTP1B expression is significantly correlated to worse survival (p=logrank).

To confirm the increased PTP1B protein expression in a larger sample group, and correlate this to clinical phenotype, PTP1B staining was performed on a tissue micro array (TMA) comprised of 455 CRC patients, of which 371 patient samples of colorectal cancer and 251 (matched) healthy tissues could be analyzed. The staining was scored according to the Allred scoring method [24] (see Figure S2). In this large cohort, we again observed a significant increase in PTP1B expression in cancer as compared to normal adjacent tissue (2.7±0.1 versus 5.6±0.1, p<0.001). For 234 patients, CRC as well as normal adjacent tissue was present on the TMA - in a paired analysis, PTP1B expression was shown to increase significantly within the same patient (2.7±0.1 versus 5.7±0.1, p<0.0001), suggesting a role for this phosphatase in the oncogenic transformation of IECs (Figure 3A).

We subsequently investigated whether increased PTP1B expression corresponds to a more severe clinical course of disease. Patients were stratified in two groups according to their PTP1B Allred score (Low < 6; High ≥ 6). This resulted in 141 (38%) patients in the PTP1B high group, and 230 (62%) in the PTP1B low group. The clinicopathological characteristics of the patient cohort and their relation to PTP1B expression levels are listed in Table 1. Interestingly, PTP1B expression is significantly correlated to well-known tumor characteristics such as TNM-stage (p=0.001), Dukes' stage (p<0.001), and tumor differentiation (p=0.038). Together this suggests that PTP1B confers a more malignant tumor phenotype in colorectal cancer. Furthermore, PTP1B is correlated to non-mucinous tumors (p<0.001), and the apoptosis markers caspase 3 and M30 (p=0.039; p=0.07, respectively). No correlation was observed between PTP1B and age, sex, tumor location, MSI-status, administration of adjuvant therapy, PIK3CA mutation, P53 expression, and KI67 expression (stained previously [22]).

**Table 1:** Patient characteristics of tissue micro array stained for PTP1B

Parameter	PTP1B Low	PTP1B high	Overall	P-value
**Number of patients**	230	141	371	
**Gender (M)**	119 (49%)	70 (51%)	189 (51%)	
**Age**	65.4	66.4	65.8	
**Location**				
Left-sided	159(71%)	94 (68%)	253 (70%)	
Right-sided	64 (29%)	44 (32%)	108 (30%)	
**TNM stadium (AJCC 5)**				
Stage I	52 (23%)	14 (10%)	66 (18%)	**0.001**
Stage II	86 (39%)	47 (33%)	133 (37%)	
Stage III	49 (22%)	51 (37%)	100 (28%)	
Stage IV	35 (16%)	28 (20%)	63 (17%)	
**Differentiation tumor**				
Well	43 (26%)	18 (15%)	61 (21%)	**0.038**
Moderate	111 (66%)	82 (69%)	193 (67%)	
Poor	14 (8%)	18 (15%)	32 (11%)	
**Dukes stadium**				
**A/B**	139 (63%)	63 (45%)	202 (56%)	**<0.001**
**C**	48 (22%)	50 (35%)	98 (27%)	
**D**	35 (16%)	28 (20%)	63 (17%)	
**Mucinous differentiation**				
No	180 (80%)	133 (97%)	313 (86%)	**<0.001**
Complete	33 (15%)	3 (2%)	36 (10%)	
Partial	12 (5%)	3 (2%)	15 (4%)	
**MSI-status**				
MSS	153 (70%)	97 (73%)	250 (70%)	
MSI-H	23 (10%)	17 (13%)	40 (11%)	
unknown	45 (20%)	18 (14%)	63 (19%)	
**Caspase 3**				
Negative	51 (23%)	16 (12%)	67 (19%)	**0.039**
Weak	118 (52%)	75 (55%)	193 (53%)	
Average	52 (23%)	41 (30%)	93 (27%)	
Strong	4 (2%)	5 (4%)	9 (2%)	
**M30**				
Low	131 (58%)	93 (67%)	224 (62%)	0.073
High	95 (42%)	45 (33%)	140 (38%)	

Corresponding to the previously observed trend in *PTPN1* levels, Kaplan Meier survival analysis revealed that high levels of PTP1B expression are significantly inversely correlated to overall survival (OS; logrank p=0.003), disease free survival (DFS; logrank p=0.001), local recurrence free survival (LRFS; logrank p=0.003), distant metastasis free survival (DMFS; logrank<0.001), and disease specific survival (DSS; 0.001) (Figure 3B–3F). Next, OS and DFS were analyzed in a multivariate analysis including the variables; sex, age at time of operation, TNM stage, dukes' stage, tumor grade, administration of adjuvant therapy, tumor location, and PTP1B status. Using backward selection, as expected, age and TNM-status were found to be independent predictors for both survival outcomes. Furthermore, PTP1B was a borderline significant independent predictor for OS, HR 1.29 (CI; 0.99-1.68, P=0.06), and a significant independent predictor for DFS, HR 1.36 (CI; 1.05-1.75, P=0.02) (Tables 2-3).

**Table 2:** Uni- and multivariate analysis for overall survival

	Univariate HR	p-value	Multivariate HR	p-value
**PTP1B**	1.471 (1.139-1.898)	**0.003**	1.286 (0.988-1.675)	0.062
**Gender (M)**	1.304 (1.044-1.629)	**0.019**		
**Age**	1.039 (1.028-1.05)	**<0.001**	1.048 (1.035-1.061)	**<0.001**
**TNM stage**				
stage 1		**<0.001**		**<0.001**
stage 2	1.386 (0.983-1.954)		1.711 (1.125-2.603)	
stage3	2.078 (1.459-2.959)		2.375 (1.539-3.664)	
stage 4	6.142 (4.199-8.985)		7.19 (4.57-11.31)	
**Dukes' stage**				
A/B		**<0.001**		
C	1.696 (1.3-2.212)			
D	4.987 (3.698-6.726)			
**Differentation**	0.96 (0.825-1.117)	0.596		
**Adjuvant therapy**	1.056 (0.957-1.166)	0.279		
**Tumor location**	1.258 (0.988-1.601)	0.063		

**Table 3:** Uni- and multivariate analysis for disease free survival

	Univariate HR	p-value	Multivariate HR	p-value
**PTP1B**	1.504 (1.172-1.931)	**0.001**	1.355 (1.048-1.752)	**0.02**
**Gender (M)**	1.289 (1.037-1.604)	**0.022**		
**Age**	1.032 (1.021-1.042)	**<0.001**	1.036 (1.024-1.048)	**<0.001**
**TNM stage**				
stage 1		**<0.001**		**<0.001**
stage 2	1.398 (0.999-1.956)		1.638 (1.088-2.449)	
stage3	2.085 (1.474-2.949)		2.169 (1.424-3.302)	
stage 4	6.171 (4.232-8.999)		6.645 (4.273-10.355)	
**Dukes' stage**				
A/B		**<0.001**		
C	1.69 (1.302-2.193)			
D	4.979 (3.694-6.71)			
**Differentation**	0.986 (0.852-1.141)	0.853		
**Adjuvant therapy**	1.036 (0.937-1.145)	0.494		
**Tumor location**	1.107 (0.872-1.405)	0.404		

### PTP1B phosphatase activity

**Figure 4:**
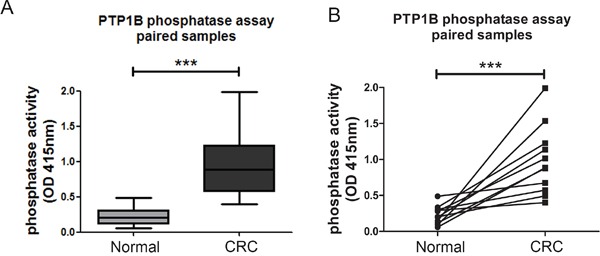
Intrinsic activity of PTP1B is increased in colorectal cancer Phosphatase activity was assessed using an immunoprecipitation based assay under “saturating conditions”. **A, B.** Quantification of intrinsic phosphatase activity of 11 paired cancer and normal adjacent colon samples, represented as means (A), or individual pairs (B). (*** P >0.001).

While PTP1B expression is increased in CRC, the more important question is whether this is followed by a corresponding increase in enzymatic phosphatase activity in these tumors. Therefore we compared PTP1B phosphatase activity in cancerous and healthy colonic tissue using an immunoprecipitation based phosphatase activity. PTP1B was precipitated under saturating conditions in order to ensure equal amounts of precipitated PTP1B protein in each sample, allowing assessment of intrinsic enzymatic activity. Freshly frozen colon cancer and normal adjacent tissues of 11 resected patients were used for this assay. PTP1B phosphatase activity was significantly increased in the cancerous tissue as compared to its control (0.98±0.47 versus 0.23±0.12 respectively, p=0.001, Figure 4A, 4B). Together this shows that not only are PTPB1 expression levels increased in CRC, the specific intrinsic activity of the enzyme is also significantly higher.

### Effect of PTP1B downregulation on cell proliferation

**Figure 5:**
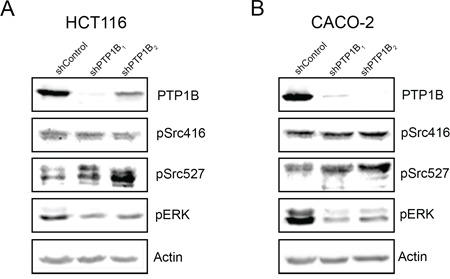
Downregulation of PTP1B interferes with cancer signaling pathways HCT116 and CACO-2 cells were lentivirally transduced with 2 different shRNAs directed against PTP1B and non-target control vector. Western blot analysis of PTP1B knockdown and control cells in HCT116 **A.** and CACO-2 **B.** cells reveals increased phosphorylation of Src Y527, and reduced phosphorylation of ERK1/2 in the knockdown cells.

**Figure 6:**
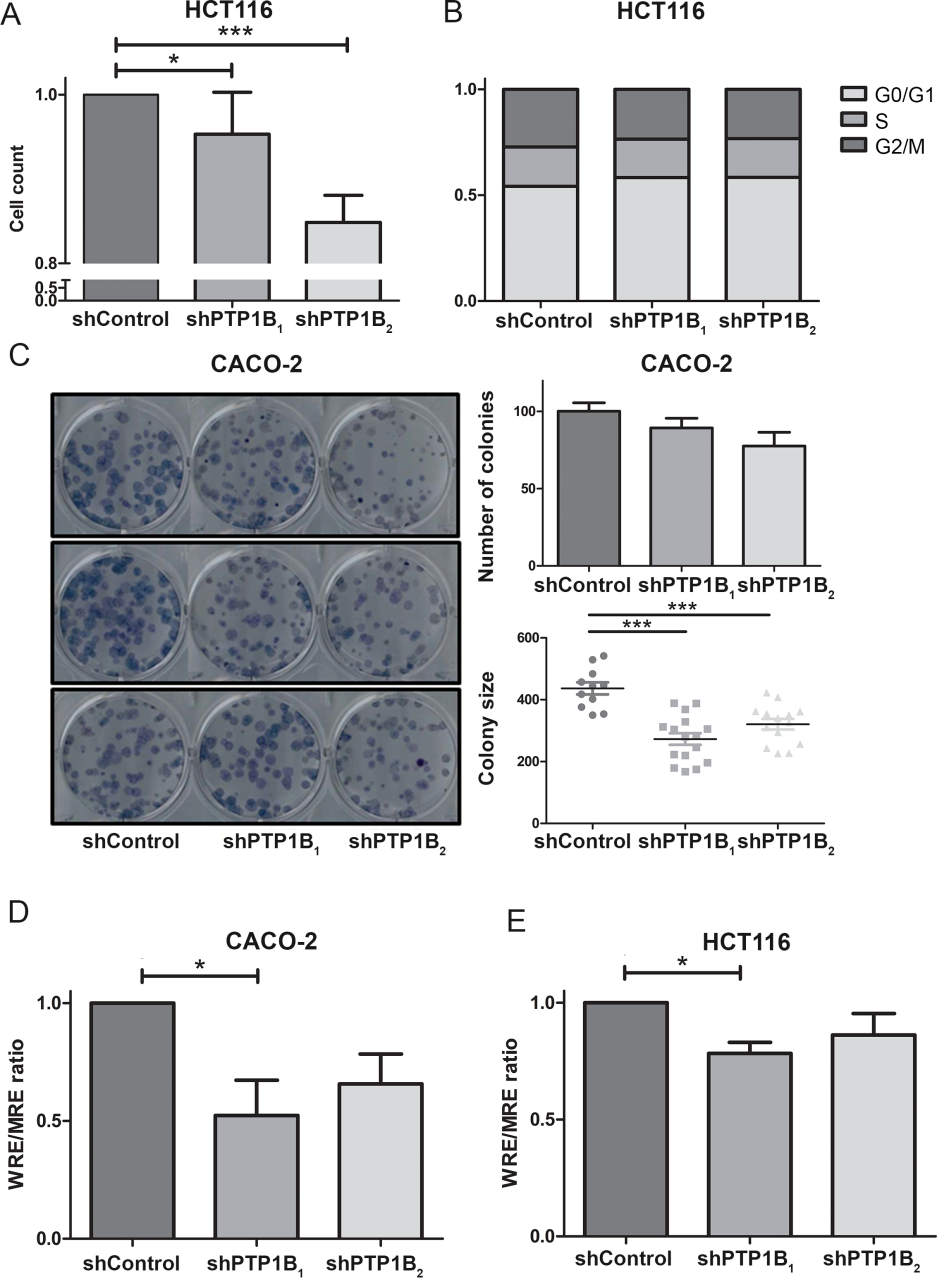
Knockdown of PTP1B reduces cell proliferation and β-catenin signaling **A.** MTT proliferation assay of HCT116 knockdown and control cells after 96 hours reveals a slight decrease in cell numbers in the knockdown cell lines (* P >0.05; *** P >0.001). **B.** Cell cycle analysis using Propidium-iodine staining of HCT116 cells followed by FACS analysis shows that PTP1B induces a slight G0/G1 cell cycle arrest, however this is not significant. **C.** Clonogenic assay of CACO-2 control and knockdown cells shows a reduced number of colonies, which are significantly reduced in size (*** P >0.001). **D, E.** β-catenin reporter assay of CACO-2 and HCT116 control and knockdown cells show reduced β-catenin signaling levels upon PTP1B knockdown.

Having established that PTP1B protein expression and phosphatase activity are increased in colorectal cancer tissues, we were interested in the cellular and molecular consequences of this phosphatase in CRC cells. Therefore, we lentivirally transfected two different colorectal cancer cell lines (CACO-2 and HCT116) with 2 different shRNAs directed against PTP1B or control vector, which resulted in stable cell lines with a significant reduction in PTP1B levels (Figure 5A, 5B upper panels). We subsequently assessed several molecular signaling cascades which in diverse cell settings have been described as targets for PTP1B (as defined by human protein reference database, http://www.hprd.org/ptms?hprd_id=01477&isoform_id=01477_1&isoform_name), i.e. the Scr kinase (Y416 and Y527) and ERK1/2 kinase. We observed that knock down of PTP1B resulted in hyperphosphorylation of the oncogenic kinase Src at the Y527 position, indicating that this residue is most likely a direct substrate for PTP1B in CRC. Phosphorylation of Y527 inactivates Src by folding it up to a closed, inaccessible conformation. In contrast, phosphorylation of residue Y416, which opens up the molecule in to an active state, was not affected, suggesting that the overall effect of PTP1B downmodulation on Src constitutes inactivation. Unexpectedly, phosphorylation of extracellular signal-regulated kinase (ERK1/2) was greatly reduced upon PTP1B knockdown (Figure 5A, 5B), indicating that this kinase is not a direct substrate of PTP1B in CRC, but is indirectly positively regulated by this enzyme. As part of the RAS-RAF-MEK-ERK pathway, ERK1/2 plays a role in the proliferation of intestinal cells [29]. Therefore we assessed CRC cell numbers using MTT and colony formation assays, which demonstrated a modest decrease in cell expansion upon reduction of PTP1B expression. Cell cycle analysis indicated tendency towards G0/G1 cell cycle arrest, although this effect was not significant (Figure 6A–6C and Figure S3A-S3C). Since up to 95% of CRCs harbor mutations in APC or β-catenin leading to constitutive activation of the Wnt/β- catenin pathway, we investigated the influence of PTP1B on this key proliferation signaling pathway. Both CACO-2 and HCT116 also have a deregulated Wnt/β-catenin, based on an APC mutation in CACO-2 cells and a β-catenin mutation in HCT116 cells. Interestingly, we observed that while these cell lines have a mutationally activated Wnt/ β-catenin pathway, downregulation of PTP1B can reduce the β-catenin signaling dosage (Figure 6D–6E). Together, these data suggest that this phosphatase stimulates the cell growth of colorectal cancer cells.

### PTP1B influences cellular migration

**Figure 7:**
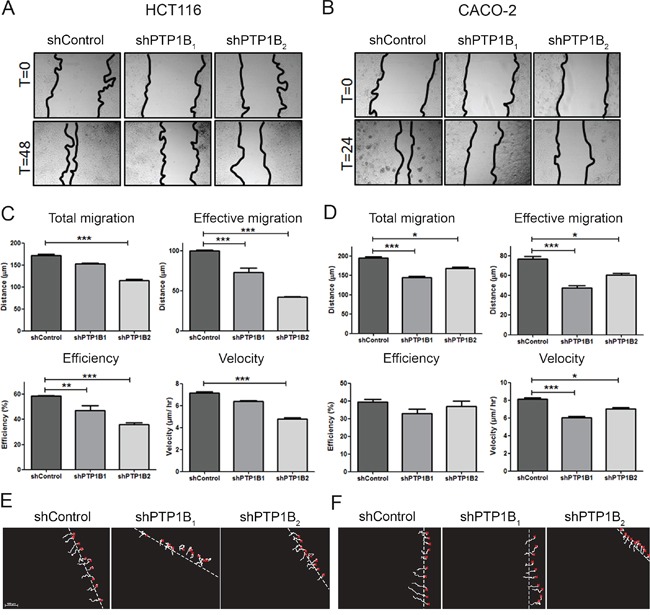
PTP1B downregulation impairs cell migration **A, B.** HCT116 and CACO-2 cell migration was measured by scratch assays, where simple scratch wounds were made using a pipet tip, and pictures are taken at 0h, 24h, and/or 48h. **C, D.** Two-dimensional migration was analyzed using a ring-barrier system. HCT116 and CACO-2 cell migration on gelatin was tracked during 24h, with locations being captured using time-lapse microscopy every 12min (x=start, line=cell track). Quantification of migrated path indicates that the total migration and velocity were significantly reduced in PTP1B knockdown cells. Effective migration is even further reduced. (D; *P<0.05; **P<0.01; ***P<0.001). **E, F.** Track diagrams of migrated path of individual cells (x=start, line=cell track).

Next, we examined the influence of PTP1B on the cell migratory capacity. Confluent plates of CACO-2 or HCT116 cells were scratched using a pipet tip, and cell migration into the wound was assessed. We observed that reducing PTP1B expression significantly impaired cell migration into the wound (Figure 7A–7B). To exclude the possibility that this effect is caused by the effect of PTP1B knockdown observed earlier on cell proliferation, we used a second approach in which the migration of individual cells is tracked. As shown in Figure 7C–7F, we again observed a significant decrease in both the total and effective migration upon PTP1B knockdown using this approach.

**Figure 8:**
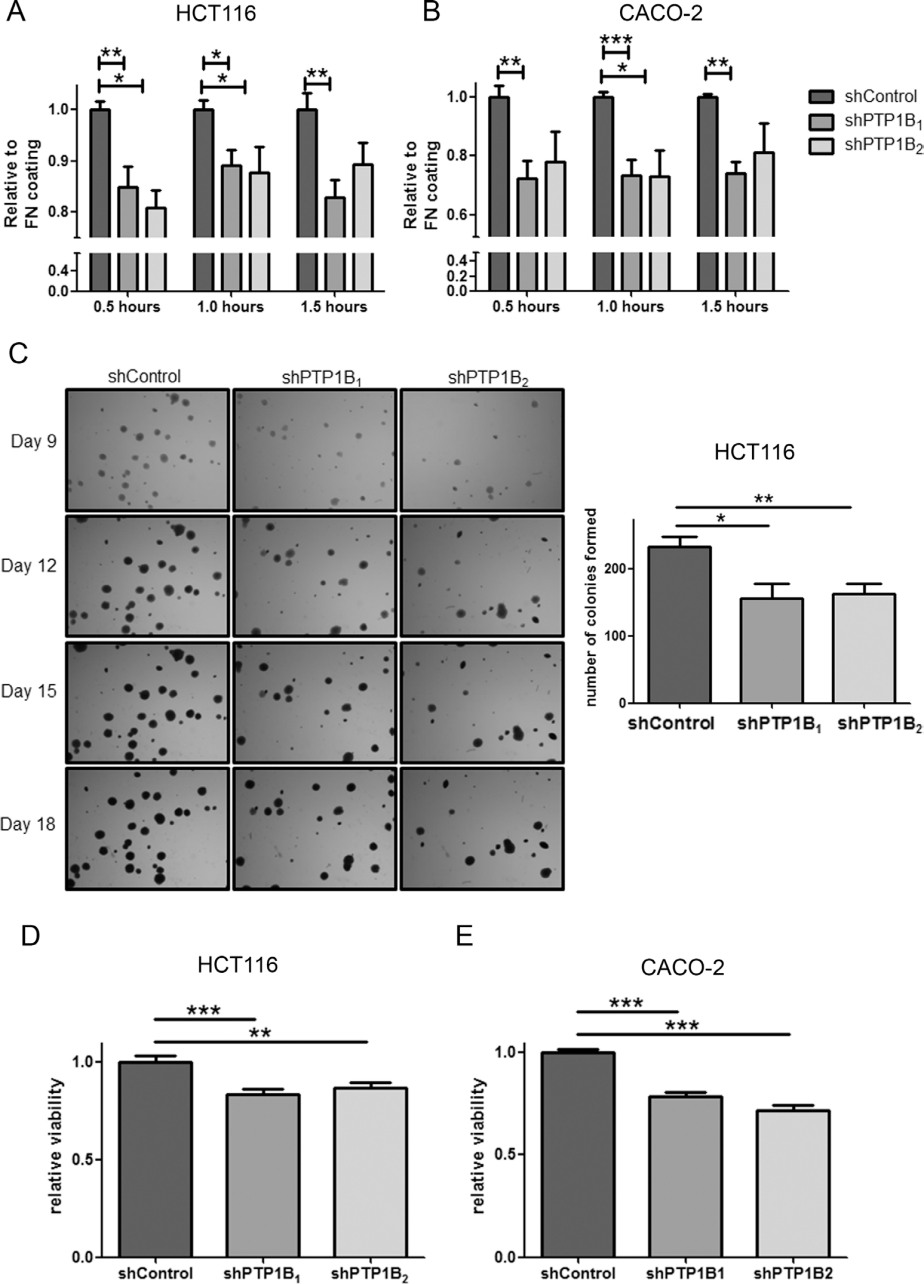
Adhesion and anoikis resistance are defected in PTP1B knockdown cells **A, B.** CRC cell adhesion was determined by MTT assay of adherent cells after indicated time points, with fibronectin (FN) coating serving as control. HCT116 and CACO-2 PTP1B knockdown cells adhere less than control cells (*P<0.05; **P<0.01; ***P<0.001). **C.** Soft-agar colony formation of HCT116 cells shows reduced ability for knockdown cells to form colonies as shown by a reduced number in colonies (*P<0.05; **P<0.01). **D, E.** Anoikis resistance assay by plating cells on poly-HEMA coated plates, showing significantly reduced cell numbers after 24 hours of culturing on these plates (**P<0.01; ***P<0.001).

Since the capacity of cells to migrate is highly dependent on their ability to adhere to the cellular matrix, we studied the adhesive capabilities of these cells. Upon PTP1B knockdown, cells are much less able to adhere to the glass surface as compared to the non-target control cells, which corresponds with the reduced migratory potential observed above (Figure 8A–8B).

### PTP1B confers anoikis resistance

In addition to migration, an important requirement for tumor cells to successfully metastasize is anoikis resistance. Anoikis is a form of programmed cell death, activated when cells detach from the extra-cellular matrix. Since metastasizing cancer cells need to survive after detachment from this matrix, they tend to become resistant to this process. Therefore we studied the effect of PTP1B knockdown on anoikis resistance by anchorage independent growth assays and cell survival in suspension cultures. Figure 8C shows colony formation of single cells embedded in soft agar, showing the potential of these cells to survive without adherence to ECM or other cells. HCT116 cancer cells easily form colonies in this assay, however the number of colonies formed upon knockdown of PTP1B is significantly reduced. The more epithelial like cell line CACO-2 is less capable of forming colonies in agar, nevertheless the PTP1B knockdown cell lines form less colonies than the non-target control cell line. Next, we further investigated anoikis resistance by culturing the cells on poly-HEMA coated plates, which prevents attachment of cells to the plate. After 24 hours we evaluated the number of living cells by MTT assay and compared this to uncoated plates as control. Knockdown of PTP1B significantly reduced viable cell numbers in suspension cultures as compared to control cells (Figure 8D–8E), again suggesting that PTP1B confers anoikis resistance in CRC.

## DISCUSSION

Protein tyrosine phosphatase 1B plays a dual role in oncogenesis depending on the tissue type. In this study we examined the role of the PTP1B in colorectal cancer. We observed a significant increase in PTP1B protein expression in primary colorectal cancer specimens, in agreement with a recent publication by Chen and colleagues [21]. Furthermore, we found that increased PTP1B leads to worse patient survival, and we showed that this increase is an independent (negative) prognostic factor for disease free survival. Importantly, in addition to an increased expression level, we found that the specific intrinsic activity of this enzyme is increased in CRC as compared to normal adjacent tissue. While the concept of phosphatases as oncogenes rather than tumor suppressors is becoming more and more accepted, few phosphatases have been shown to act in a tumor promoting way in CRC. Recently, we have described an oncogenic role for the low molecular weight protein tyrosine phosphatase (LMWPTP) in colorectal cancer, which is significantly upregulated in CRC, and follows a step-wise increase from normal cells to adenoma and carcinoma [23]. Similarly, the stomach-associated phosphatase 1 (SAP-1) promotes the oncogenic potential of intestinal epithelial cells, and is overexpressed in colorectal tumor tissues [30]. Most extensively studied is the phosphatase of regenerating liver-3 (PRL-3), which was shown to be upregulated in up to 100% of CRC liver metastasis cases, and overexpressed in primary tumors [31, 32]. However, none of these studies report on the intrinsic activity of the phosphatase studied. Even though PTP-targeting agents have not yet reached the clinic, most of the developed compounds target the catalytic activity of the enzymes [33], therefore our finding on the enzymatic activity is of significant importance. With the recently reported promising results of the PTP1B-inhibitor Trodusquemine in breast cancer [19], our data provide further rationale for the use of these kinds of PTP1B activity inhibitors as treatment modality for colorectal cancer.

After our initial finding that PTP1B expression and activity are significantly overexpressed in colorectal cancer, we evaluated the effect of PTP1B *in vitro*. As mentioned earlier, depending on the cell type, PTP1B can have both oncogenic and tumor suppressive functions. Potentially, these differences arise as a consequence of different modulation of downstream signaling modules in these tumors. ERK phosphorylation is often found to be upregulated in tumors, and as ERK has been identified as a direct substrate of PTP1B, our findings may seem counterintuitive. However, as Src is a known activator of ERK, it is likely that reduced ERK phosphorylation observed upon PTP1B knockdown is a direct consequence of the inactivation of Src in these cells. Such would be in line with earlier reports showing that PTP1B, Scr and ERK may be linked in lung, gastric and breast cancer [34–37]. Furthermore, PTP1B may affect ERK independently of Src, as PTP1B-deficient fibroblasts were shown to display decreased Ras-activity, resulting in reduced ERK phosphorylation [38].

In the current study, we show that the altered signaling induced by PTP1B expression, which includes aberrant canonical Wnt/β-catenin and Src/Erk signaling culminates in an enhancement of CRC metastatic potential by increasing proliferation, migrational activity, and anoikis resistance in these cells. Although the impact of PTP1B on anoikis has not been described before, this corresponds well to an earlier report in prostate cancer, which shows a significantly reduced cell migration upon PTP1B downregulation or chemical PTP1B inhibition [16]. However, in contrast to our findings, it has been described in hepatocellular carcinoma that low PTP1B expression predicts a poor prognosis in this tumor type, and that this low expression is correlated to high nuclear – thus activated – β-catenin expression [39]. Similarly, in P53-deficient mice, knockout of PTP1B makes these mice more susceptible to the development of B-cell lymphomas compared to PTP1B WT mice, however the exact molecular mechanism affected remains to be elucidated [14].

Since we observed a significant upregulation of PTP1B protein and *PTPN1* mRNA levels in colorectal cancer, the question rises how this upregulation occurs. Using the cbioportal TCGA database, we observed that PTP1B is frequently overexpressed in cancer due to amplification of the 20q13 chromosomal region. This region has been identified a long time ago as frequently amplified in breast cancer [40], and later also in prostate and colorectal cancer [41, 42]. Interestingly, in all these cancer types amplification of this locus is linked to a more invasive or metastatic tumors, which corresponds well to the phenotype we observe in our PTP1B downregulated cells. In the TCGA dataset we also found that *PTPN1* upregulation is related to chromosomal amplification, which could be related to direct *PTPN1* promotor activation. In the promotor region of *PTPN1*, multiple binding sites for transcription factors have been described. They include transcription factors such as Sp1, Sp3, Egr-1 [43], NF-kB [44], and HIF [45], which can all be activated as part of malignant processes, and can therefore result in increased *PTPN1* transcription. However, this would not directly account for the observed increase in intrinsic phosphatase activity observed in CRC tissue. PTP1B activity may be reduced by oxidation [46], phosphorylation [47], or sumoylation though the E3 ligase PIAS1 [48], but which of these mechanisms contribute to the increased phosphatase activity observed here remains to be elucidated.

In summary, we show that protein tyrosine phosphatase 1B is overexpressed in colorectal cancer on protein and mRNA level, which results in a significant reduced survival. Moreover, we show that the enzymatic activity is greatly increased in the colorectal tumors. This results in a more malignant phenotype by enhancing proliferation, migration, and anoikis resistance of intestinal epithelial cells. Together this shows that targeting the activity of this phosphatase could be a very promising novel treatment option for CRC, and a step forward in the fight against colorectal cancer.

## MATERIALS AND METHODS

### Gene expression profiles

The Oncomine database tool (www.oncomine.org) was used to analyze *PTPN1* mRNA expression from microarray data. Briefly, the *PTPN1* gene was queried in the database and the results were filtered by selecting colorectal cancer studies reporting on the *PTPN1* gene (Reporter ID: 202716). P-values for each group were calculated using students' t-test. Standardized normalization techniques and statistical calculations are provided on the Oncomine website. Furthermore, the Cancer Genome Atlas (TCGA) dataset was analyzed using cbioportal (www.cbioportal.org), correlating *PTPN1* expression levels to methylation status and clinical data available at this website.

### Patients and tumors on tissue micro array

As proof of principle, we started by performing immunohistochemistry on formalin fixed paraffin embedded (FFPE) colorectal tissue specimens collected from the Erasmus Medical Center department of pathology from 9 low grade dysplasia (LGD) patients, 5 high grade dysplasia (HGD) patients and 7 adenocarcinoma (CRC) patients. Ulcerative colitis (n=8) served as controls. Thereafter, the staining was extended to a tissue microarray, consisting of a cohort of 470 colorectal cancer patients treated with surgery for their primary tumor in the Leiden University Medical Center (LUMC) between 1991 and 2001 [22]. Clinicopathological and follow-up data were collected retrospectively from hospital records and the oncology database. This research was performed according to the code of conduct for responsible use. Patient records were anonymized and de-identified prior to analysis according to national ethical guidelines (“Code for Proper Secondary Use of Human Tissue,” Dutch Federation of Medical Scientific Societies). Patients with multiple simultaneous colonic tumors were excluded from the analysis (n=21). The entire study cohort consisted of 455 patients.

### Immunohistochemistry

The FFPE tissue sections and TMA were immunohistochemically stained for PTP1B (PTP1B antibody, sc-14021, Santa Cruz Biotechnologies, Dallas, Tx, concentration 1:50) as described before [23]. The slides were scored for the percentage of positive epithelial cells as well as intensity and PTP1B staining was subsequently quantified using the a scoring system based on the Allred score for immunohistochemistry [24]. This score is a combination of a proportion score (% positive IEC), and intensity score. The proportion score was defined as; 0 for 0% IEC, 1 for 1-10% IEC, 2 for 10-30% IEC, 3 for 30-50% IEC, 4 for 50-80% IEC, and 5 for 80-100% positive IEC. Intensity was scored as negative (0; very low or no staining), weak (1+; intensity just above background level), intermediate (2+; clearly visible at low magnification) and strong (3+; striking at low magnification). Together this results in an Allred score between 0-8. For each patient on the TMA, at least two tissues cores were available. Patients were stratified into two groups according to the PTP1B expression as defined by Allred score <6 (low) and >6 (high). This cut-off was arbitrarily chosen to result in two comparable groups.

### Cell lines and transfections

HCT116 and CACO-2 colorectal cancer cells and HEK293T cells were purchased from ATCC (Manassas, USA) and cultured in Dulbecco's Modified Eagles Medium (DMEM, Lonza, Basel, Switzerland), supplemented with 10% fetal bovine serum (Sigma-Aldrich, St. Louis, USA). All cell cultures were supplemented with 100 U/ml penicillin, 100 mg/ml streptomycin (Life technologies, Bleiswijk, NL), and propagated at 37°C in a 5% CO2 humidified atmosphere. Using a lentiviral system, stably transfected PTP1B knockdown cells were created. HEK293T cells were transfected with 2μg of shRNA (Sigma-Aldrich, St. Louis, USA), together with lentiviral vectors (1μg of pVSVG, 1μg of pREV and 2μg of pMD) in a 6 well plate using PEI transfection Reagent (DNA: PEI ratio, 1:5). Non-targeting shRNA was used as a control. Transfected cells were selected using puromycin (2μg/ml, Sigma-Aldrich, St. Louis, USA)

### Western blotting

Western blotting was performed as described previously [23].

### Cell viability assay

Cell viability was assessed using a colorimetric MTT (3-(4,5-Dimethylthiazol-2-yl)-2,5-diphenyltetrazolium bromide assay as described previously [23].

### Scratch migration assay

Scratch assay was performed as described previously [23].

### Adhesion assay

Adhesion assay was performed as described previously [23].

### 2D migration assay

Cell migration was analyzed using the “ring barrier system” as described previously [25].

### Clonogenic assay

Clonogenic cell survival assay was performed to assess proliferation potential. In a 6-well plate, a single cell suspension (250 cells per well) was added to each well. After two weeks, single colonies were fixed and stained by haematoxylin, after which colonies were counted and measured using the Nikon Nis Elements^®^ for windows software. Each assay was performed three times in triplicate.

### Soft agar assay

Anchorage independent colony-forming capacity was assessed using soft-agar growth. Sterile electrophoresis grade agarose was dissolved in warm culture medium at a final concentration of 1.5% and 0.5% (w/v). In a 6-well plate, a single cell suspension (1000 cells per well) was taken up in a liquid 0.5% agarose solution at 40°C and directly poured on the already solidified 1.5% bottom layer. After cooling down to RT 2 mL standard culture medium was added to cover the agarose layers. Every other day the liquid medium was refreshed. Starting from day 9 after seeding, images were taken and the colony diameter (pixels) was measured using the Nikon Nis Elements^®^ for windows software. Each assay was performed three times in triplicate.

### Anoikis resistance assay

Anoikis resistance was assessed using poly-HEMA (Poly 2-hydroxyethyl methacrylate; Sigma-Aldrich, St. Louis, USA) coated plates. 96 well plates were coated with 20mg/ml poly-HEMA in 95% ethanol and left to evaporate for 24 hours. After washing the plates, an equal number of cells were loaded. After 24 hours the amount of viable cells was determined using MTT assay. Uncoated wells were used as loading control.

### PTP1B immunoprecipitation and phosphatase assay

To quantify the intrinsic phosphatase activity, freshly frozen tumor and adjacent normal tissue blocks from resected patients material were homogenized with a tissue blender and then lysed in 500ul of Lysis Buffer (20mM HEPES, pH7.7 with 2, 5mM MgCl2, 0, 1mM EDTA, 1mM PMSF, 1mM DTT, and protease inhibitor cocktail tablet) on ice for 2h. After clarifying by centrifugation, protein concentration was measured and equal amounts of total protein were used for the immunoprecipitation. Enough protein was used to ensure that after precipitation, residual PTP1B protein was left in the lysate, thereby ensuring that the limiting factor in the precipitation are the beads and antibodies, and guaranteeing that equal amounts of PTP1B are precipitated from each sample, regardless of differences in PTP1B expression in the samples. Pre-cleared protein extracts were incubated overnight at 4°C under rotation with antibodies against PTP1B (Santa Cruz Biotechnologies, Dallax, Tx). A/G-Sepharose beads were added to cell homogenates and incubated for 2h at 4°C. After washing, the precipitate was resuspended in acetate buffer (100 mM pH 5.5) and immediately used for enzymatic assay. The PTP activity was measured using 20mM p-nitrophenilphosphate as a substrate. After 1h incubation at 37°C, plates were measured using a spectrophotometer (OD 405nm).

### β-Catenin reporter assay

β-Catenin reporter assays were performed by transfecting HCT116 and CACO-2 cells with either the Wnt-responsive element-luciferase reporter or a mutant-responsive element-luciferase variant. Tk-Renilla was used as a control for transfection efficiency. 24h after transfection, luciferase activities were measured using the dual-luciferase reporter assay system (Promega, Madison, WI, USA) and a LUMIstar luminometer according to the manufacturer's protocol.

### Statistical analysis

Statistical associations between expression of PTP1B (as categorical variable) and continuous clinico-pathological parameters (age at the time of diagnosis) were tested using Student's t-test, and categorical parameters (Gender, Tumor location, TNM-stage, Tumor differentiation, Dukes' stage, Mucinous differentiation and MSI status) using Pearson's χ2 test.

To determine whether PTP1B expression was predictive for overall survival, disease free survival, or disease specific survival, we used univariate Cox regression with following covariates; age at diagnosis (as continuous variable), gender, tumor location, TNM-stage, tumor differentiation, Dukes' stage, adjuvant therapy use, and PTP1B status (as categorical variables). For multivariate analysis, we used stepwise backward selection of the covariates. The proportionality assumption for PTP1B positive versus negative cases was visually assessed in Kaplan–Meier curves. P-value <0.05 was considered significant. All statistics were performed using SPSS 21 (SPSS, Chicago, IL, USA).

For the *in vitro* experiments groups were compared using a students' T-test and statistical analyses were performed using the Graphpad Prism 5.0 software package for Windows. A two-tailed p-value <0.05 was accepted as statistically significant. Images were composed using Adobe Photoshop CS6.

## SUPPLEMENTARY FIGURES


