# Supplementary material for: Increased PTP1B expression and phosphatase activity in colorectal cancer results in a more invasive phenotype and worse patient outcome
Source: Oncotarget. 2016 Mar 1;7(16):21922–38. doi: 10.18632/oncotarget.7829 (PMC5008334; doi:10.18632/oncotarget.7829)
Supplement: Supplementary file 1 [file oncotarget-07-21922-s001.pdf]

SUPPLEMENTARY FIGURES

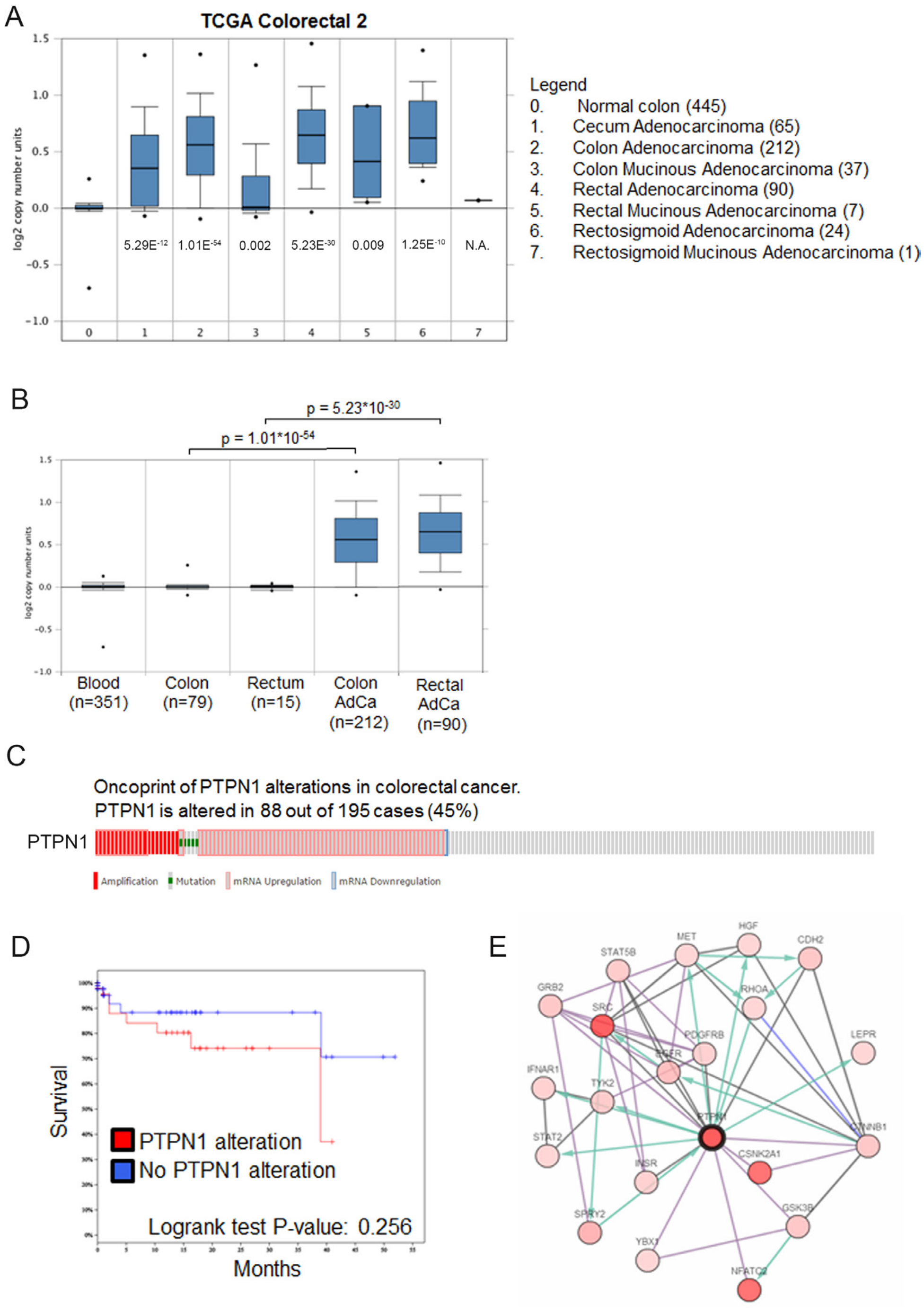

Supplementary Figure 1: PTPN1 is altered in colorectal cancer. Analysis of TCGA colorectal 2 datasets using the online tools; oncomine and cbioportal. **A.** *PTPN1* expression is increased in all subsets of colorectal cancers as compared to normal colon. **B.** Expression of *PTPN1* in colon adenocarcinoma or rectal adenocarcinoma compared to healthy tissues shows a significant increase in cancerous tissues. **C.** Oncoprint of *PTPN1* in colorectal cancer from cbioportal, shows an alteration of *PTPN1* in 45% of the CRC cases, with frequent amplifications and mRNA upregulations. **D.** Kaplan meier analysis of patients with alterations in *PTPN1* (red) and without alteration (blue), shows a trend towards worse survival in altered patients, however patient numbers are low. **E.** Pathway analysis of *PTPN1* reveals a network of oncogenes such as Src, EGFR and STAT5.

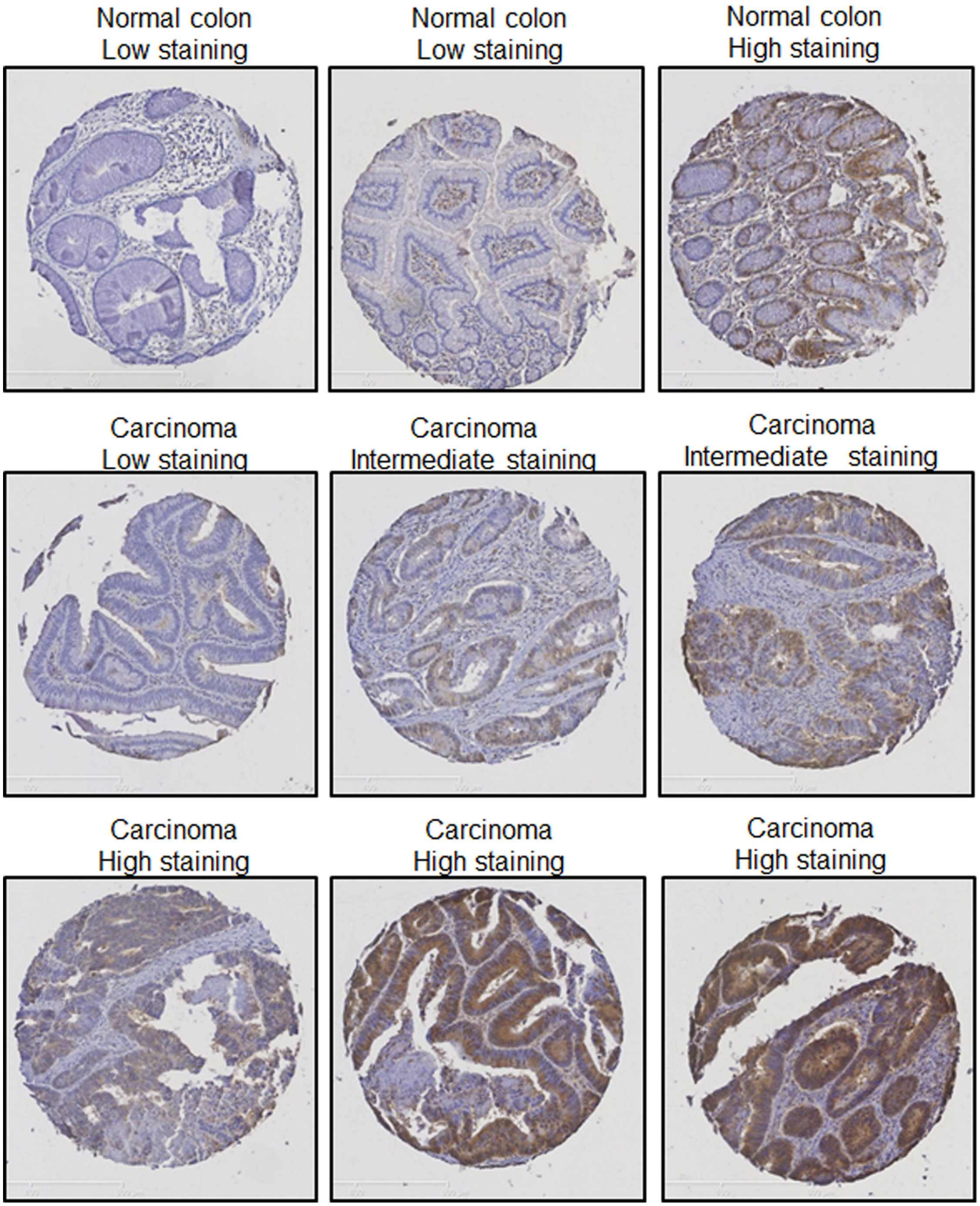

Supplementary Figure 2: Examples of tissue micro array cores stained for PTP1B. Cores of normal colonic tissue or colonic cancer tissue, showing a wide variety of staining patterns both in the normal as well as the cancerous tissues. Differing in the intensity of the staining, as well as in percentage cells that stained positive.

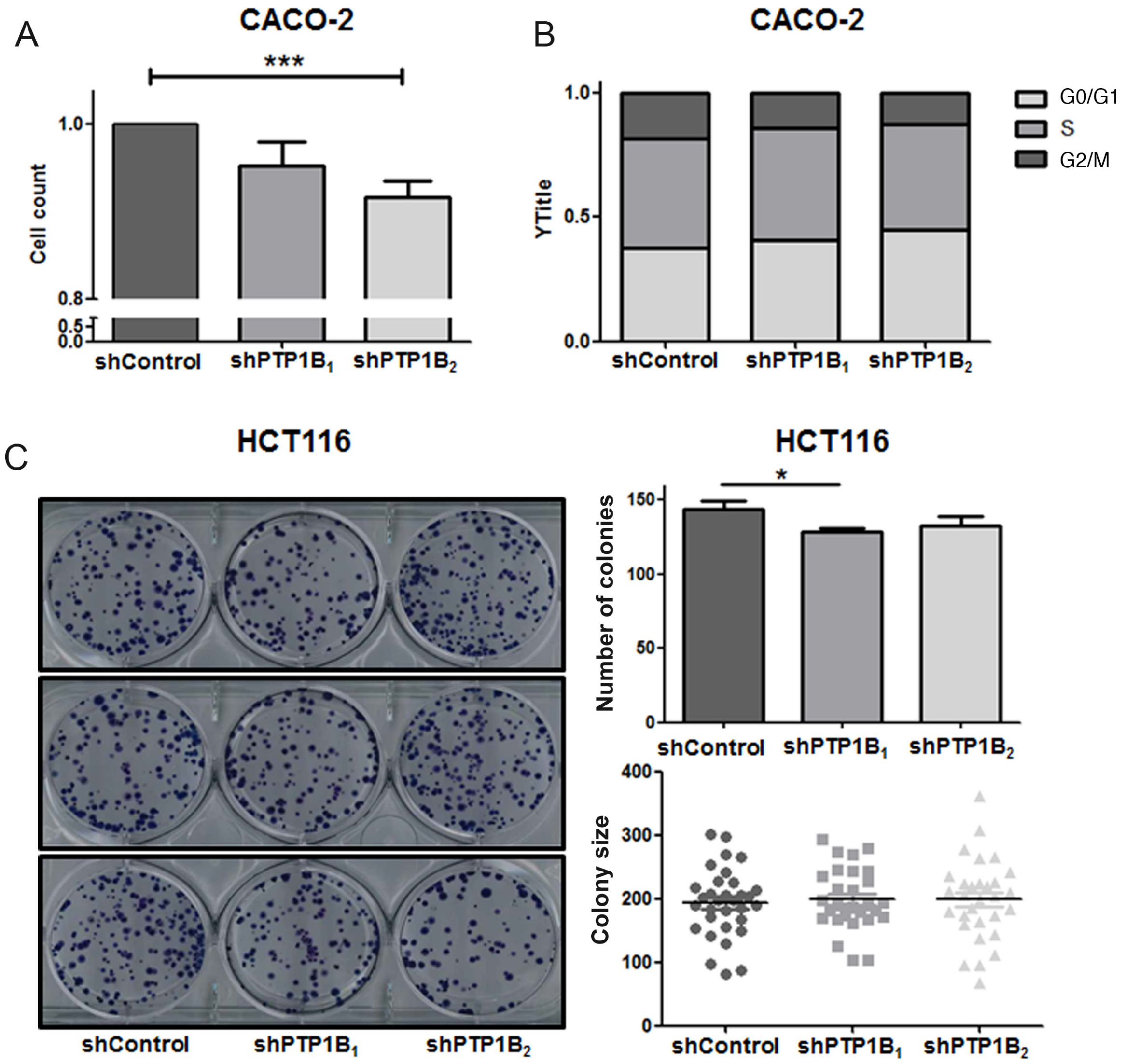

Supplementary Figure 3: Knockdown of PTP1B reduces cell proliferation. A. MTT proliferation assay of CACO-2 knockdown and control cells after 96 hours reveals a slight decrease in cell numbers in the knockdown cell lines (*** P >0.001). **B.** Cell cycle analysis using Propidium-iodine staining of HCT116 cells followed by FACS analysis shows that PTP1B induces a slight G0/G1 cell cycle arrest, however this is not significant. **C.** Clonogenic assay of HCT116 control and knockdown cells shows a reduced number of colonies (* P >0.05), and no differences in colony size.
